# Cystic Degeneration of the Chorion

**Published:** 1878-10

**Authors:** H. L. Harrington

**Affiliations:** Warren Co., Ills.


### Clinical Reports.

### NOTES FROM PRIVATE PRACTICE.

*Cystic Degeneration of the Chorion.*

On July 12th, 1877, I saw in consultation, Mrs. C——, aged 30, medium size; sanguine temperament. She gave the following history: married ten years; two children living, the youngest aged four years; one supposed abortion at third month, about two years before; general health good until recently. In March her menses did not appear, and she supposed she had taken cold; did not consider herself pregnant; menses did not re-appear in April; from the first she suffered from almost persistent nausea and vomiting; was treated by various physicians for "biliousness" and other vague disorders. Early in May, she began flowing, and later profuse hæmorrhage occurred and continued at intervals of a few days until the above date, the discharge consisting of blood and at times a little "watery fluid." About the middle of June her legs began to swell and the abdomen to enlarge, and she continuously lost flesh and strength. Her last physician had for some time been administering active diuretics and drastic cathartics; also tincture of iron. On examination I found her very anæmic; scarcely a vestige of color in cheeks or lips; emaciated; tongue pale and flabby; appetite poor; no nausea; abdomen enlarged and fluctuating; legs enormously enlarged, pitting upon pressure; suffering from dyspnœa especially when lying down. Auscultation revealed a very distinct anæmic murmur over base of heart and carotids. I at once proposed to the doctor that we examine the uterus; to this he objected, thinking that it was a "passive hæmorrhage." I then recommended a continuation of the iron with tonics and nutritious diet; to discontinue diuretics and purgatives, and, since the uterus could not be examined, to await farther developments. At 8 o'clock p. m., July 26th, I was hastily called in consultation with this same physician to see Mrs. C., who at one o'clock that day had commenced having labor pains and profuse flooding, which continued during the afternoon. About one hour before my arrival, a large mass had been expelled from the womb, which nearly filled an ordinary chamber, and which on examination proved to be a vesicular mole. Its appearance is well illustrated in the figure on page 567, Thomas' Diseases of Women, third edition. Quite a quantity of cysts still remained; these I removed and administered fluid extract of ergot and whisky freely, the latter in cream.

It seemed almost impossible for her to recover from the effects of such a prolonged and profuse drain of the vital fluid, but with the exception of what might be termed hæmorrhagic fever which followed reaction, she gradually improved, and in a few weeks was entirely well. The rare occurrence of such cases, is my apology for presenting this. In conclusion I would urge the *absolute necessity* of examining the uterus when a patient comes to us with the above symptoms. How many wives and mothers may have been sacrificed to the ignorance or indifference of the attending physician?

H. L. Harrington, M. D.

Warren Co., Ills., August 29th, 1878.

